# Emotion Regulation in Pediatric Obsessive-Compulsive Disorder and Related Interventions: A Scoping Review

**DOI:** 10.3390/children12040400

**Published:** 2025-03-21

**Authors:** Shivali Sarawgi, Rachel E. Mathews

**Affiliations:** 1 Cincinnati Children’s Hospital Medical Center, Cincinnati, OH 45229, USA; 2 College of Medicine, University of Cincinnati, Cincinnati, OH 45221, USA

**Keywords:** pediatric OCD, emotion regulation, intervention, scoping review

## Abstract

Background/Objectives: Maladaptive emotion regulation (ER) and emotion dysregulation (ED) have long been associated with obsessive-compulsive disorder (OCD) as etiological and maintaining factors. Despite building interest in the field along with ancillary research into “rage OCD” (likely an example of ED), targeting the relationship between OCD and ER/ED has been understudied in pediatric OCD populations. The aim of this review was to elucidate the current state of the literature regarding ER/ED, its relationship to pediatric OCD, and related interventions. Methods: A scoping review examined how ER/ED are related to OCD, particularly in pediatric populations, and the efficacy of interventions to affect ER/ED for youth, with a focus on youth diagnosed with OCD. Results: A total of 182 publications were reviewed. While not always consistent, a majority of studies found a significant relationship between measures of ER or ED and OCD broadly, as well as with specific OCD symptom dimensions. A number of previously existing interventions, adaptations of those interventions, and newly presented interventions were found to affect ER/ED in youth; however, few studies have effectively targeted ER/ED for pediatric OCD, specifically. As such, the mechanism of change is not well understood. Conclusions: Findings from this review suggest that the increasing focus on ER/ED in pediatric OCD is warranted and in need of continued research. ER/ED can be effectively changed by interventions in youth, but the role ER/ED-change plays in pediatric OCD symptom improvement remains unclear. Implications for future study are examined.

## 1. Introduction

Identifying factors contributing to psychopathology in pediatric populations represents an essential step in prevention and early intervention. Given the chronicity of various mental health concerns, prevention and early intervention during childhood or adolescence may be particularly important. Emotion dysregulation (ED) has long been identified as a transdiagnostic factor involved in the etiology and maintenance of a wide range of psychopathology, including obsessive-compulsive disorder (OCD), e.g., [1,2,3]. Generally, ED refers to deficits in the ability to reliably and appropriately modulate the expression, duration, and/or intensity of the emotional experience. Similarly, emotion regulation (ER) is thought to be a variably automatic to deliberate activation of the goal to influence the emotional trajectory [4], including type of emotion, time-course, expression, and experience. Unsurprisingly, multiple forms of psychotherapy (particularly within the cognitive–behavioral framework) have directly addressed ED or the intrinsically related construct of emotion regulation (ER), as attention has shifted to addressing transdiagnostic processes in treatment. However, deeper understanding of the role ER/ED plays in psychopathology and as a target intervention has been obfuscated by the lack of definitional clarity and standard measurement.

Indeed, the state of the literature does not present a cohesive and consistent theory of ER or ED, e.g., [5,6]. Many studies focus on either ER or ED; see Blader, Garrett, and Pliszka for discussion and review [7]. Some models and studies conceptualize ER as strategies to *adaptively* impact and respond to emotions, while others have conceptualized various regulatory strategies as *maladaptive*. For instance, Gross indicates a number of ER strategies including situation selection, attentional deployment, thought suppression, and response modulation as various ER strategies [6]. However, situation selection and attentional deployment could well be used in a maladaptive manner (e.g., avoidance), and thought suppression has consistently been identified as maladaptive in conjunction with various psychopathology [8,9,10]. This is further complicated by differences in the utility/harm of some regulatory approaches depending on the implementation and specific psychopathology (e.g., use of cognitive reappraisal prior to engagement in exposures, which may decrease the effectiveness of inhibitory learning [11]). Some symptoms of specific psychopathology have further been labeled as a regulator strategy, such as hair-pulling in trichotillomania. Additionally, some have conceptualized ED as merely defined by difficulty with adaptive ER skills, while others conceptualize ED as inappropriately experienced (e.g., intensity and reactivity) and expressed emotions [5,12]. There are also various models of comprising factors for ER/ED, with a lack of clarity and distinction [13]; for instance, definitional overlap between alexithymia and emotional clarity/awareness, e.g., [14], distress tolerance as a separate construct versus a factor of ER, e.g., [15], and the conceptual relationship with executive functioning, e.g., [16].

There is an expansive research base delineating the utility of appropriate emotion regulation development. Indeed, research by Riediger and Bellingtier [17] suggests that ER “not only contributes to developmental adaptation, it is also a developmental phenomenon itself” (p. 4). The current literature suggests that ER skills are essential for developmental adjustment across the lifespan [17], with specific ER competencies predicting improved academic achievement, increased prosocial behaviors and social functioning, and reduced risk for psychopathology [18,19]. Robust development of ER skills is also predictive of enhanced mental and physical health, improved interpersonal relationships, and increased occupational success in adulthood [17,20,21,22]. As such, the development of ER skills in one’s formative years, with continued honing of such skills through adolescence and young adulthood, is an essential process from a developmental perspective. Specific internal and external factors, such as developing neurobiological structures, cognitive capabilities, and family relationships, also impact ER at different life stages [17]. Moreover, given the numerous developmental dimensions upon which ER is loaded, appropriate development of ER skills is a public health concern.

Given the proposed transdiagnostic properties of ER/ED, these constructs have been routinely studied in various pediatric populations as well. As indicated above, ER skills can be important protective factors, with one study demonstrating that greater ER capacity is preventative for mental and physical health disorders and may lead to more successful social development [23]. Conversely, ED has been demonstrated to significantly increase impairment for those with behavioral health concerns, in youth populations specifically [24]. As interest in ER/ED grows for pediatric populations, more attention has been paid to the ability to successfully intervene on ER/ED. Reviews have found various psychosocial interventions to demonstrate effectiveness and pharmacotherapy as providing some added benefit [25]. Despite this increased attention, less has been explored regarding the effectiveness of these interventions to meaningfully impact ER/ED in pediatric OCD populations and/or the development of novel interventions for youth with OCD and ED. Targeting ER/ED may have significant implications for youth with OCD given the importance of enhancing distress tolerance and inhibitory learning through ERP. In addition, compulsions in OCD, and similar behaviors in OC and related disorders (OCRDs), have often been posited to serve an emotional regulatory purpose, e.g., [26,27]. It is worth noting that the role of ER/ED has been investigated further for adult OCD populations with promising findings. Improving ER skills, such as acceptance of emotions and emotional awareness (established ER skills [5]), have regularly been found to improve treatment outcomes for individuals with OCD [28,29,30], and increasing emotional awareness may improve the efficacy of ERP through linguistic processing [11].

Examining ED and related interventions for pediatric OCD is of further interest given the regularity with which caregivers have historically noted rage attacks. Rage attacks in OCD are a distinct clinical phenomenon that is well established in the existing literature [31,32,33,34]. Rage attacks have been defined as temper tantrums or aggressive outbursts that often occur in response to disruption of the child’s OCD-related symptoms/behaviors. These episodes may include coercive–disruptive behaviors, such as forceful efforts to impose symptom accommodation on family members [35]. Research by Storch et al. [36] found that 54.7% of youth within a clinical sample demonstrated rage attack symptoms within the past week, while 53% demonstrated significant rage episode within the past month. The existing literature suggests that the majority of rage attacks consist of verbal aggression, which is often directed at parents and siblings. However, rage attacks associated with OCD can escalate to include physical aggression and may be directed at individuals outside of the immediate family unit. Fortunately, additional research has ascertained that the frequency of rage attacks tends to diminish as the youth with OCD approaches adulthood. The extant literature has identified that OCD rage attacks are associated with a number of negative outcomes, including more severe clinical presentation, greater functional impairment, increased family accommodation, and weaker response to treatment [35,36,37,38,39]. Research by Peixoto and Marques [40] indicates that the presence of rage attacks perpetuates long-term frequency and severity of OCD symptoms, as well as an increased level of impairment.

## 2. Materials and Methods

**Figure 1:**
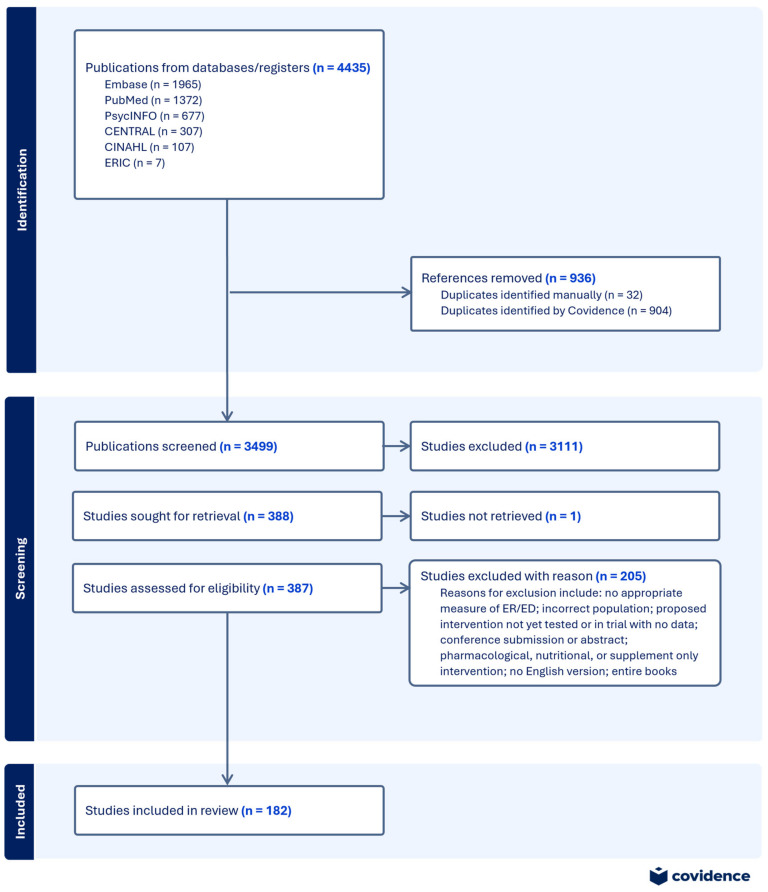
PRISMA-SCR flow diagram.

This paper provides a scoping review, reported according to the PRISMA extension guidelines for scoping reviews (PRISMA-ScR) [41]. This review was conducted utilizing the following stages [42,43]: (1) identify the research question, (2) identify relevant studies, (3) select studies, (4) chart data, and (5) collect, summarize, and report the results. The review protocol has not been registered; the PRISMA-ScR flow diagram can be found in Figure 1.

### 2.1. Identify the Research Question

Broadly, this review sought to discover what is known about the presence of ED in pediatric OCD as well as examine available evidence-based interventions targeting ED in pediatric OCD. Scoping reviews aim to synthesize broad evidence, particularly for emerging areas of study [41,44]. Related questions that informed our search and analysis included the following: how does ED affect presentation, treatment engagement, and family response in OCD across the lifespan as well as what evidence-based interventions have been found to be efficacious in targeting ER/ED in pediatric populations.

### 2.2. Identify Relevant Studies

Comprehensive literature searches were carried out utilizing the following databases: PubMed, PsychINFO, Cumulative Index to Nursing and Allied Health Literature (CINAHL), Embase, CENTRAL, Education Resources Information Center (ERIC). The search was limited to publications between 2005 and 2025 with all searches conducted in December 2024. Peer-reviewed journal articles, case studies, book chapters, and dissertations were included in the search, excluding conference abstracts and entire books where no individual chapter was identified. Publications not available in English and studies utilizing nonhuman subjects were also excluded. Additionally, empirical studies that did not include a measure of ER, ED, or some empirically identified factor (e.g., irritability, cognitive flexibility) or if the only measure was neurophysiological were excluded from the review. Lastly, only publications providing information on ER/ED in the context of OCD or OC-symptoms (regardless of age) and publications providing information on interventions in pediatric populations were included. Specific keyword searchers utilized to identify articles are contained in Appendix A, Table A1.

### 2.3. Study Selection

Covidence (www.covidence.org; accessed on 17 December 2024), a web-based platform designed to screen and extract information from publications, was used to import, organize, and manage all considered publications. A search of PubMed yielded 1372 publications, PsycInfo led to 677 publications, CENTRAL resulted in 306 publications, Embase identified 1965 publications, ERIC yielded 7 publications, and CINHAL resulted in 107 studies. Nine hundred four duplicates were identified by Covidence, and 32 duplicates were identified and removed manually. Exclusion criteria were applied at the title and abstract level; two authors each screened all article abstracts to ensure 100% agreement of articles that would be included in the full-text article review. Three hundred eighty-eight publications were identified for full-text review that were subsequently examined by both authors utilizing inclusion/exclusion criteria. The full text for one study was not available for retrieval; the authors of the study did not respond to requests for the full text. This selection process yielded 182 studies, as can be observed in the flow diagram presented above. A quality review of these studies was not conducted, as this is generally considered outside the purview of a scoping review.

### 2.4. Charted Data and Results Collation

Covidence was again utilized to export data from the full text of the selected publications using a standardized template across authors that was tested prior to its use; data charting was completed by one author, independently. Study information extracted from each publication was entered into Excel, which was used to organize, synthesize, and understand information and themes across the studies. The following variables were coded during data extraction: title, year of publication, geographical location, publication type, aim of publication, sample characteristics (including age range, clinical status, and symptom qualifiers), whether studies stratified data based on demographics, structure of ER/ED measure (informant report, behavioral, physiological, or measuring an empirically identified aspect of ER/ED only, e.g., irritability), target of intervention, and whether ED/ER served as a moderator or mediator relative to OCS or OC-severity. All variables of interest and coding specific information are contained in Appendix A, Table A2.

## 3. Results

The review found n = 182 publications meeting the review criteria, across 109 journals, 4 book chapters, and 4 dissertations. The journal with the greatest number of publications meeting criteria was the *Journal of Obsessive-Compulsive and Related Disorders* (n = 23). Publications were found from all continents outside of Antarctica; more specifically, publications were identified from countries in Africa = 2, Asia = 16, Australia/New Zealand = 18, Europe = 49, the Middle East = 12, North America = 84, and South America = 1. Only 4 of these 182 publications were published before 2010, with only 15 more published before 2015. Another 50 were published between 2015 and 2019; more than 60% (n = 112) of the publications were published in 2020 or later. Notably, few of these publications (n = 4, including one dissertation) examining ER/ED and OC-symptoms in pediatric populations were identified prior to 2015, suggesting that interest in targeting these factors in pediatric OCD has gained momentum only in the last decade. A minority of studies (n = 18) reported findings after disaggregating data by sex or by stratifying data by sex or gender. The majority of studies that did so (n = 11), compared groups based on sex or groups based on gender (at least one study compared on both sex and gender) and found no significant difference by group. This was analyzed in the present review according to more broadly accepted definitions of sex and gender. Quality and bias assessment are considered beyond the purview of a scoping review, e.g., [41,44], and as such are not further illustrated here. However, we recognize the likelihood of their presence in the primary sources, given the lack of disaggregated or stratified data as related to any demographic variable. Studies comprised experimental designs (n = 56), quasi-experimental designs (n = 42), case reports and series (n = 5), and other observational studies (i.e., cross-sectional and correlational; n = 63). Also included in the selected publications were n = 16 systematic reviews, meta-analyses, narrative reviews, and/or review-based book chapters linking ER/ED to OCD.

### 3.1. Diagnoses

Among the total publications found, studies examining the efficacy of interventions for pediatric populations with symptoms of ER/ED as an outcome spanned a variety of diagnoses, some within the same publication, n = 13. Typically, however, a publication focused on one primary diagnosis of interest, with n = 12 publications targeting children/adolescents with ASD, n = 8 with a mood disorder, n = 4 with a disruptive behavior psychopathology, n = 3 with primary ADHD, n = 6 with weight or eating concerns.

The majority of the publications focused on OCD/OCS utilized clinical populations. Additionally, n = 20 OCD-related publications reported on the effects for symptom measures only, without confirming an OCD diagnosis; four of those publications involved pediatric populations. Another 19 publications presented information on obsessive-compulsive-related disorders (OCRD; n = 9 hoarding, 8 body-focused repetitive behaviors, and 2 body dysmorphic disorder publications); which were all specific to adult populations and were primarily experimental in nature.

### 3.2. Emotion (Dys)regulation

Of note, many publications differed with regard to a focus on ER versus ED, and definitions of both ER and ED varied across the literature contained in the review, e.g., [45]. Measures of ER or ED also differed significantly across publications. The majority of publications included a self-, parent, or teacher report of ER or ED (n = 162). In empirical studies, 60 utilized the Difficulties with Emotion Regulation Scale (DERS; [5]), 30 included the Emotion Regulation Questionnaire (ERQ; [46]) and/or the ERQ for Children and Adolescents (ERQ-CA; [47]), and five incorporated the Cognitive Emotion Regulation Questionnaire (CERQ; [48]). The most commonly used measure in pediatric empirical studies was the DERS (n = 18), as was true for empirical studies in adult populations (n = 42). The DERS and ERQ were also the most commonly observed measure of ER/ED for studies specifically examining the relationship with OCD or OCS. A total of 18 publications (8 that included pediatric samples) utilized both informant report measures as well as a behavioral and/or physiological measure of ER. Three papers utilized only a naturalistic/behavioral and/or physiological measure of ER. Physiological measures of ER included heart rate variability (HRV) and skin conductance, while examples of a naturalistic/behavioral measure includes the Go/No-Go Computer Task [49] and eye-gaze duration.

Variability was noted in how authors attributed certain measures or features to ER, self-regulation, cognitive regulation, social regulation, or separate constructs. For instance, distress tolerance as measured by the Distress Tolerance Scale (DTS) [50] was noted as a facet of ED only in studies by Macatee and colleagues [51,52]. Some studies utilized the Behavior Rating Inventory of Executive Function (BRIEF) [53], which is a measure of executive functioning (EF), specifically for the emotional control subscale, which was utilized as a measure of ER as opposed to a factor of EF, e.g., [54,55]. There was also a mixture as to whether papers referred to ER when describing both adaptive and maladaptive strategies versus just adaptive, which mirrors the history of confusion in the way the literature defines these terms. Lastly, cognitive reappraisal and suppression from the ERQ or ERQ-CA were regularly utilized in OC studies and largely identified reappraisal as an adaptive strategy, e.g., [56,57,58].

### 3.3. Pediatric Emotion (Dys)regulation and Related Intervention

A number of publications (n = 82) focused on exploring ER/ED either as a primary or secondary outcome variable in the context of intervention; in accordance with our broader questions and inclusion criteria, 78 of these studies were in pediatric populations and addressed a wide range of psychopathology and related constructs. The majority of these studies tested an existing intervention (e.g., dialectical behavior therapy, cognitive therapy, mindfulness, neurofeedback) or a modified version of the intervention (e.g., motivational interviewing plus dialectical behavior therapy). Interventions were largely found to have utility in impacting ER/ED, e.g., a trial of Unified Protocol-Child Version in children with anxiety [59], though this was not consistent, e.g., a trial of dialectical behavior therapy in adolescents with bipolar disorder [60]. Many studies examined ER/ED as a primary outcome, but even where ER/ED were secondary outcomes, few studies investigated the mediational or moderation effect of ER/ED on effectiveness of treatment, symptom reduction, or health improvement; five publications found a significant mediation or moderation effect.

### 3.4. OCD and Emotion (Dys)regulation

Eighty-three publications focused on exploring the general relationship between OC-symptoms and ER/ED, including reviews and book chapters. Sixty-three publications presented data from clinical populations with OCD diagnoses, and 20 publications presented data on OC-symptoms in undiagnosed samples. While inconsistent, data from these publications in addition to data presented in intervention studies suggest general difficulties in emotion regulation for individuals with OCD as well as a poorer emotion regulation associated with OC-symptoms. The relationship between ED and OCD exists even when controlling for other variables, as is seen in research by Yap and colleagues [61], who found that the DERS significantly predicted OCD symptom severity even when controlling for age, sex (report of male/female according to the primary source), depression, and anxiety. Specific associations between OC-symptom dimensions and particular features of ER/ED were examined in several studies. A study by Khosravani, Ardestani, Bastan, and Malayeri [45] yielded significant associations among specific obsessive-compulsive dimensions (checking/doubting, obsession, mental neutralizing, and ordering) with non-acceptance of emotions, checking/doubting one’s own emotional awareness, and ordering as it relates to accessing appropriate emotion regulation strategies.

There was some inconsistency in the relationship between emotional awareness/emotional clarity and OCD, with some publications suggesting little to no relationship and some finding there to be greater problems with emotional understanding and awareness, e.g., [13,61,62,63]. One study posited that obsessive-compulsive symptomatology may be associated with limited understanding of emotions, as well as negative reactivity to experiences with emotion [27]. See et al. [13] observed that individuals with OCD are more likely to demonstrate weaker emotional awareness than healthy controls. Additional research suggests poorer emotional awareness has been found to be associated with greater frequency and intensity of OCS [64]; this research, in conjunction with Yazici and Yazici [63], also suggests an association between weaker emotional clarity and OCD/OCS. Another study by Stern et al. (2014) [27] discovered that obsessive-compulsive symptom distress was positively associated with weaker emotional understanding and awareness, as well as heightened fear of both positive and negative emotions. They posited that obsessive-compulsive symptomatology may be associated with limited understanding of emotions, as well as negative reactivity to experiences with emotion. Alternatively, Salamon, Augsburger, and Dan-Glauser [65] found that high levels of OCS did not correlate with emotional awareness in a nonclinical sample. Similarly, Yap and colleagues [61] showed that significant relationships of OCD/OCS with emotional awareness and clarity may lose significance when controlling for or moderated by anxiety and depressive symptoms. Other research observed similar findings for emotional awareness [45,62].

One additional finding was the association found between OCD/OCS and suicidality, with most publications in this review demonstrating a positive association. Notably, ER/ED appear to influence this relationship. For instance, research by Mikonowicz and Tull [66] identified a significant association not only between OCS severity and ED but also ED and suicide risk; further, there was a significant indirect relation of OCS through ED with suicide risk. Another study found that the effect of childhood maltreatment on suicidal ideation in OCD patients was mediated, in part, by adaptive emotion regulation skills [67].

#### 3.4.1. OCD, Emotion (Dys)regulation, and Interventions

Intervention studies involving adult samples with OCD, again, largely showed an ability to impact ER/ED with existing interventions or modifications to existing interventions. Common intervention strategies observed in the publications examined include cognitive or cognitive–behavioral strategies, ERP, acceptance-based interventions, and mindfulness skills. A study by Allen and Barlow [28] supported the use of psychoeducation related to the nature of emotions and consequences of emotional avoidance, as well as emotional awareness training and exposure to non-specific (also described as clinically irrelevant) emotional cues. Findings of this study suggested that participants demonstrated a reduction in emotional avoidance and thought suppression, with acceptance of thoughts and feelings being areas of emphasis. One trial of the Unified Protocol was also found to successfully reduce both disorder-specific psychopathology as well as improve emotion regulation skills; however, neither mediating nor moderating effects of change in ER difficulties were found for improvement in symptoms [68]. As with the broader intervention literature for ER/ED, neurofeedback was examined in adult OCD patients; however, unlike findings in pediatric learning, regulatory strategies before neurofeedback did not appear to affect scores of ER for these patients.

#### 3.4.2. Pediatric OCD and Emotion (Dys)regulation

Fifteen publications (n = 11 of which were experimental/observational studies) examined ER in youth with OC-symptoms, including 11 publications examining children and adolescents with clinical diagnoses of OCD (Table 1). Similar findings were noted in youth populations to those observed in adult populations. However, several studies examining children and adolescents further included age-specific measures including family accommodation and externalizing behaviors. As with the broader lifespan literature, data from pediatric populations demonstrated some inconsistencies. ER or ED were often found to be related to, be affected by, or to affect OC-symptoms (Table 2). This varied somewhat on the measures used, particularly measures of ER. For instance, McKenzie and colleagues [55] identified a high emotional control and low emotion control (EC, as measured by the BRIEF) group based on a median split of youth receiving CBT with ERP. Those in the low EC group demonstrated greater OCD severity, received more family accommodation, and had higher internalizing and externalizing symptoms. They were less likely than those in the high EC group to achieve response or remission of OC-symptoms. In a study examining ER through a broader measure (ERQ-CA), no significant changes were observed throughout the course of treatment for youth with a primary diagnosis of OCD [69].

As with the adult population, publications identified by this review examined interventions including cognitive or cognitive–behavioral strategies as well as acceptance and mindfulness skills (Table 3). Five studies examined ER/ED in the context of interventions for pediatric OCD populations (less than 7% of the intervention studies identified in this review); these included a measure of OCS and/or provided interventions to OCD samples. One randomized-control study specifically targeted ER/ED skills, including thought suppression and acceptance, and the effects on symptoms [70]. This study demonstrated that youth instructed to engage in acceptance of thoughts over thought suppression experienced a greater decrease in distress; this was a nonsignificant trend. They further found a greater decrease in subjective thought frequency, again at trending significance with a large effect size. Another intervention study found [55] that in addition to main effects of intervention on ER/ED and/or OCS, greater emotional lability/negativity was associated with greater family accommodation and externalizing symptoms, while adaptive ER was negatively correlated with externalizing symptoms. Their measure of ED (a measure of emotional lability and negativity) moderated the relationship between OC-severity and family accommodation. Of note, these intervention studies appeared primarily based in first and second wave cognitive behavioral therapies (CBT; e.g., examining outcomes for youth in a CBT with ERP treatment or examining constructs such as thought suppression and reappraisal). One study suggested that, after CBT, youth with an OCD diagnosis did not demonstrate changes in expressive suppression and cognitive reappraisal, unlike youth diagnosed with an anxiety disorder [69]. A number of the studies identified as addressing ER/ED in broader youth samples (described above) utilized other treatment modalities such as third wave CBT strategies (e.g., dialectical behavior therapy, mindfulness) and newly developed treatment packages comprising various evidence-based elements, e.g., [71,72]. Given the publications found in this review, it is difficult to determine whether some of these interventions, determined to be efficacious in other populations, are efficacious for pediatric OCD populations as well.

## 4. Discussion

The purpose of this review was to examine the role of ER/ED in pediatric OCD as well as interventions for this population. This was achieved through gathering information about the state of the literature concerning ER/ED in OCD and ER/ED-related interventions available for pediatric populations. ER/ED as a mediator or moderator of intervention effects was also of interest for any intervention study in which ER/ED was not the primary outcome. Interestingly, few studies investigated the effect of specifically targeting ED as a part of treatment for pediatric OCD. Moreover, a similarly limited number of the included studies provided empirical testing of an intervention for pediatric OCD, with results examined in the context of ED. One promising study [74] utilized an intervention for pediatric OCD in which ER was targeted as a part of the overall intervention (positive family interaction therapy); however, this study was excluded at the full-text level due to having no measure of ER/ED. Despite demonstrating significant symptom improvement, the lack of an ER/ED outcome measure made it difficult to determine whether effects were truly related to any change in ER and if ER played a mediating or moderating role in OC-symptom reduction. Results from the review indicate ER/ED and OCS can be successfully targeted by intervention, but results were mixed for whether ER/ED played a role in OC-symptom change. Further, numerous studies demonstrated that interventions can efficaciously decrease ED or maladaptive ER strategies and/or increase adaptive ER skills. Given the lack of studies directly examining the effects of intervention on ER/ED and/or OCS/OC-severity, more interventional research is necessary to determine whether existing treatments are sufficient in addressing both domains, how manipulating ER/ED can impact OCD treatment, and how, if at all, specific ER skills should be taught or highlighted as a part of treatment.

Previously identified concerns regarding the lack of consensus in defining ER/ED were observed in the literature, across ages and clinical group/status. Nevertheless, the findings from the present review continue to support the idea that ER/ED, as a transdiagnostic factors, are also of relevance for individuals with OCD, including pediatric populations. This included findings of specific facets of ER/ED associated with overall OCD/OCS as well as with specific OC-symptom dimensions. However, these findings were not consistent. These studies were primarily limited to adult populations as well. Given the mixed findings, understanding the relationship between dimensions of OCD and factors of ER/ED in pediatric populations may augment any related intervention research, particularly in the context of OCD-specific treatment considerations [75]. Some theories, such as inhibitory learning, have suggested that utilizing ER skills such as emotional awareness and labeling as well as cognitive reappraisal based on learnings from ERP are beneficial for treatment. However, these theories also caution against the use of other emotion regulation skills, including physiological relaxation strategies and pre-exposure cognitive reappraisal that alter the emotional experience (which is often what is meant by reappraisal in the ER literature [46]). Interestingly, in both adult and pediatric OCD intervention studies, there were few data on the effect of ERP on ER/ED or augmentation of ERP with ER skills; instead, much focus was placed on cognitive strategies despite previous findings of OCD patients have decreased access to appropriate ER skills and frequency of positive affect [61,64]. Our review demonstrates these ideas need to be tested more completely.

### Limitations

Limitations of this review include the inclusion of a limited number of databases utilized in the search. Search terms may not have been maximally utilized, such as terms referencing specific ER skills or factors of ER/ED, such as irritability, cognitive reappraisal, and emotional awareness. Inclusion of these terms may have yielded richer data about the association of ER/ED with pediatric OCD as well as greater knowledge of, or more specified implications for, relevant interventions. Additionally, the present review did not examine ER/ED-related interventions for adult populations and could only examine interventions for ER/ED across various pediatric populations; given the dearth of intervention-focused research related to ER/ED in pediatric OCD populations, comparison of existing interventions between OCD and other clinical populations could not be discussed. Some studies have found differing levels of occurrence, and differential impacts, based on gender or sex for the variables of interest in this review. The current literature base, as identified from the publications in this review, does not thoroughly examine gender differences or differences in sex assigned at birth when presenting data on variables of interest for youth with OCD. Gender and sex were often poorly defined (e.g., % female, boys) or used interchangeably within the same publication; researchers are encouraged to clearly define all variables, including demographic variables, to prevent biases and misrepresentation of data. As such, this review was unable to determine if sex assigned at birth or gender played a role in the relationship between ER/ED and OCD.

## 5. Conclusions

Much is known about various aspects of ER and ED, as well as the specific nature of their relationship to OCD. Various interventions from differing theories of ER/ED are successful in affecting these factors for pediatric populations. More research is needed to understand the utility of existing ER/ED interventions in pediatric OCD, the relationship between ER/ED and treatment efficacy using gold-standard ERP, and the role that affected change in ER/ED plays in OC-symptoms improvement. Practically, these findings suggest practitioners would do well to consider the role of ER/ED when providing therapy for youth with OCD, particularly in relation to maladaptive regulation that would interfere with treatment and in relation to family accommodation. Further implications from this review’s findings include the need for clinicians, patients, and families to formulate a clear understanding of emotion regulation strategies, such as thought suppression, cognitive reappraisal, and acceptance. These are difficult concepts that may require a level of metacognition not easily accessible to youth and their families. Nevertheless, comprehension of these concepts may lead to significantly different outcomes. Additionally, capitalizing on the link between ER and ED may provide another avenue for symptom change in pediatric OCD, a relevant method of creating readiness for change, or a method of preventing interference from other symptoms while engaging in ERP. As a transdiagnostic factor, addressing ED during treatment may also lay the foundation for comorbid symptom change and overall maintenance of gains. Continued research may further elucidate whether ERP sufficiently impacts ER/ED and, if necessary, how best to incorporate adaptive ER skills in line with current theoretical approaches to ERP (e.g., inhibitory learning model). Compared to the wide range of intervention models and treatment packages identified for other pediatric populations, those examined in pediatric OCD populations had limited variability in approach. Researchers are encouraged to test a greater variety of evidence-based strategies that target ER/ED directly or that have been shown to lead to related improvements. This would provide further knowledge into the role of targeting ER/ED for youth with OCD in addition to allowing for comparison across clinical populations. Broader pediatric interventions further utilize a variety of modalities, not observed in the pediatric OCD literature, which may be more beneficial than those observed in this review. Any future investigation of ER/ED in pediatric OCD would benefit the field by broadening attention for factors beyond reappraisal and suppression, particularly given the potential for harm in both strategies. Current studies for pediatric populations also do not delineate the “active ingredients” for efficacious treatment, which are essential to providing efficacious care; rigorous research into individual strategies is warranted.

Research should continue to strive toward conceptual consensus of ER and ED as constructs, as well as the factors comprising them. The lack of conceptual clarity and the variety in operational definitions for these terms prevents generalization and translation of intervention strategies across domains of psychopathology. This is particularly unfortunate given the transdiagnostic nature of ER/ED that would lend itself well to transdiagnostic intervention, a method of increasing efficiency of treatment. Lastly, despite lay interest in rage attacks in OCD, few studies in this review provided significant discussion about these concerns in relation to ER/ED, leaving further gaps between subjective experiences and concerns and empirical knowledge. Future research into rage attacks, in addition to methods of presenting this information and related recommendations to families, is much needed. This scoping review demonstrates the increased interest and significant opportunities for growth in the field’s understanding and utilization of interventions affecting ER/ED in pediatric OCD.

## Figures and Tables

**Table 1:** Publication counts for pediatric OCD and ER/ED publications (n = 15).

Category	Count
Experimental study	1
Quasi-experimental study	4
Other observational study	6
Review (article or book chapter)	4
Clinical OCD sample	11
Informant report measure of ER/ED	11
Studies finding a relationship between OCD/OCS and ER/ED	10

**Table 2:** Matrix of specific relationships for pediatric OCD and ER/ED.

	Adaptive ER/ ER Skills				Maladaptive ER/ ED		
		Acceptance	Reappraisal	Other		Suppression	Other
**OCD diagnosis/ severity/OCS**	X (4)		X (2)	X	X	X (2)	X (3)
**OCD distress**		X				X	
**Obsessional** **beliefs**	X					X	
**Contamination** **/washing**	X					X	X (2)
**Checking**	X					X	
**Obsessing**			X	X		X	X
**Ordering/** **arranging**						X	X
**Neutralizing**			X	X		X	X
**Doubting**			X	X			X
**Hoarding**			X	X			X
**Family** **accommodation**	X				X		

Note: variables as defined by the study authors. Number found in parentheses delineates the number of studies reporting this relationship.

**Table 3:** Publication characteristics for pediatric OCD and ER/ED intervention studies.

	Publication Type	Study Design	Population (Clinical Profile)	Measure of ER/ED	Intervention
Harrison (2011) [70]	Dissertation	Experimental	OCD diagnosis and other clinical population	Author generated	Thought suppression vs. acceptance
Ogle (2020) [73]	Dissertation	Quasi-experimental	OCD diagnosis and other clinical population	CEFI	CBT
Wei, et al. (2020) [58]	Journal Article	Quasi-experimental	OCD diagnosis	ERQ	Intensive treatment (E/RP-based)
McKenzie et al. (2020) [55]	Journal Article	Quasi-experimental	OCD diagnosis	BRIEF-EC	Intensive CBT with E/RP
Knowles & Tolin (2024) [69]	Journal Article	Quasi-experimental	OCD diagnosis and other clinical population	ERQ-CA	CBT

Note: CEFI = Comprehensive Executive Functioning Inventory—parent report; ERQ = Emotion Regulation Questionnaire; BRIEF-EC = Behavior Rating Inventory of Executive Function-Emotional Control subscale; ERQ-CA = Emotion Regulation Questionnaire for Children and Adolescents; CBT = Cognitive Behavioral Therapy.

## Data Availability

The data analyzed in this review were derived from previously published data. The data that support the findings of this study are available from the respective authors of these publications and may be subject to restrictions.

## Abbreviations

The following abbreviations are used in this manuscript:

OCD	Obsessive-compulsive disorder
OC	Obsessive-compulsive
OCS	Obsessive-compulsive symptoms
ER	Emotion regulation
ED	Emotion dysregulation
ERP	Exposure with response prevention
OCRDs	Obsessive-compulsive and related disorders
ACT	Acceptance and Commitment Therapy
CENTRAL	Cochrane Central Register of Controlled Trials
ERIC	Education Resources Information Center
CINHAL	Cumulative Index to Nursing and Allied Health Literature
DERS	*Difficulties with Emotion Regulation Scale*
ERQ	*Emotion Regulation Questionnaire*
ERQ-CA	*Emotion Regulation Questionnaire for Children and Adolescents*
CERQ	*Cognitive Emotion Regulation Questionnaire*
DTS	*Distress Tolerance Scale*
BRIEF	*Behavior Rating Inventory of Executive Function*
HRV	Heart rate variability
EC	Emotional control
PRISMA	Preferred Reporting Items for Systematic reviews and Meta-Analyses
PRISMA-ScR	PRISMA for scoping reviews
ASD	Autism Spectrum Disorder
ADHD	Attention-Deficit/Hyperactivity Disorder
CEFI	*Comprehensive Executive Functioning Inventory- parent report*
BRIEF-EC	*Behavior Rating Inventory of Executive Function-Emotional Control subscale*
CBT	Cognitive Behavioral Therapy

## Appendix A

### Appendix A.1

Table of search terms. These two search phrases were utilized for each database.

**Table A1:** Search strategy used in all databases.

((emotion dysregulation) OR (emotion regulation)) AND ((obsessive-compulsive) OR (obsessive compulsive))
((emotion dysregulation) OR (emotion regulation)) AND (pediatric) AND ((intervention) OR (treatment))

### Appendix A.2

List of variables that were coded from the Covidence extraction.

**Table A2:** Coded variables of interest.

Variable	Description
Title of work	[Text]
Year published	[Numerical]
Location	Africa, Asia, Asian subcontinent, Australia/New Zealand; Europe, Middle East, North America, South American
Type of publication	Journal, book, university of dissertation
Aim of study	[Text]
Study design	Experimental, quasi-experimental, case report, other observational, review, book chapter
Data stratification	Yes/No
Population (age group)	Pediatric, adult, lifespan
Population (clinical status, symptoms of interest)	OCD diagnosis, OCS measured (clinical status not confirmed), other clinical group, nonclinical group without OCS measured
ER/ED (type of measure)	Informant report, physiological, behavioral
ER/ED (specific measure)	[Name of any measure utilized e.g., DERS, eye-gaze]
General result summary	[Text]
Specified relationship between OCD/OCS and ER/ED	[Text with data]
Type of intervention	New or new adaptation of an existing intervention [Yes/No]
ER/ED as a moderator or mediator	[Text] if reported

Note: OCD = obsessive-compulsive disorder, OCS = obsessive-compulsive symptoms, ER = emotion regulation, ED = emotion dysregulation, DERS = Difficulties in Emotion Regulation Scale.
